# Metastatic Merkel Cell Carcinoma Masquerading as Multiple Immune-Related Adverse Events

**DOI:** 10.1155/2020/8890845

**Published:** 2020-09-30

**Authors:** Farees Saqlain, Sophia Z. Shalhout, Kevin S. Emerick, Tomas G. Neilan, Tatyana Sharova, David Michael Miller

**Affiliations:** ^1^Harvard Medical School, Boston, MA, USA; ^2^Department of Medicine, Division of Hematology/Oncology, Massachusetts General Hospital, Boston, MA, USA; ^3^Department of Otolaryngology, Massachusetts Eye and Ear Infirmary, Boston, MA, USA; ^4^Department of Otolaryngology, Harvard Medical School, Boston, MA, USA; ^5^Department of Medicine, Division of Cardiology, Massachusetts General Hospital, Boston, MA, USA; ^6^Department of Dermatology, Massachusetts General Hospital, Boston, MA, USA

## Abstract

Merkel cell carcinoma is a rare cutaneous neuroendocrine carcinoma with a high rate of regional and distant metastasis and mortality. Here, we report a novel case of Merkel cell carcinoma which presented as a primary lesion to the left cheek with regional lymph node involvement and was treated with pembrolizumab and radiation. Widely metastatic disease eventually revealed on autopsy clinically mimicked immune-related organ insult leading to management with immunosuppressants. The patient also had a biopsy-confirmed immune-related cutaneous adverse event during admission. The case highlights a rare circumstance in which disease progression masqueraded as multiple immune-related end-organ adverse events. Contribution of on-target anti-PD-1 toxicity remains a possibility.

## 1. Introduction

Merkel cell carcinoma (MCC) is an aggressive primary cutaneous neuroendocrine carcinoma with risk factors including lighter complexion, age over 50, ultraviolet exposure, and immunosuppression [1]. The disease has a particularly high rate of locoregional and distant metastasis. A review of 9387 cases from the National Cancer Database showed that 65% of patients presented with local disease, 26% presented with nodal spread, and 8% presented with distant metastasis, with respective 5-year overall survival estimates of 51%, 35%, and 14% [2]. Common sites for distant metastasis include the distant lymph nodes, liver, lung, skin and subcutaneous tissue, and bone [3, 4]. Since 2017, immune checkpoint inhibitors have been approved by the FDA for medical management of metastatic disease, with the potential to cause a diverse array of immune-related adverse events (irAEs). Clinically significant hepatotoxicity, for example, is known to occur with pembrolizumab in a small subset of patients, typically 2 to 6 cycles after treatment initiation [5]. Here, we describe a novel case of regionally metastatic MCC managed with pembrolizumab and radiation, with distant, multisite progression initially masquerading as multiple end-organ immune-related adverse events. While biopsy supported attribution of concomitantly presenting dermatitis as an irAE, autopsy revealed metastatic disease to the liver, heart, muscle, and bone with little accompanying inflammation.

## 2. Case Presentation

**Figure 1:**
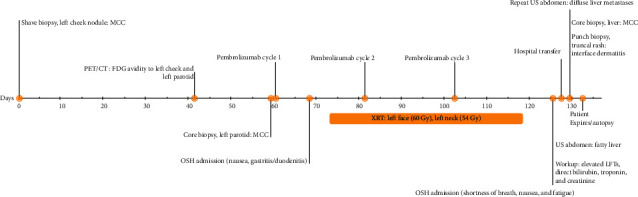
Patient clinical course. A timeline was provided beginning at day 0, set to the initial histological Merkel cell carcinoma diagnosis of the left cheek nodule. Abbreviations: MCC, Merkel cell carcinoma; PET/CT: positron emission tomography/computed tomography; FDG: fluorodeoxyglucose; OSH: outside hospital; XRT: radiation therapy; US: ultrasound; LFTs: liver function tests.

**Figure 2:**
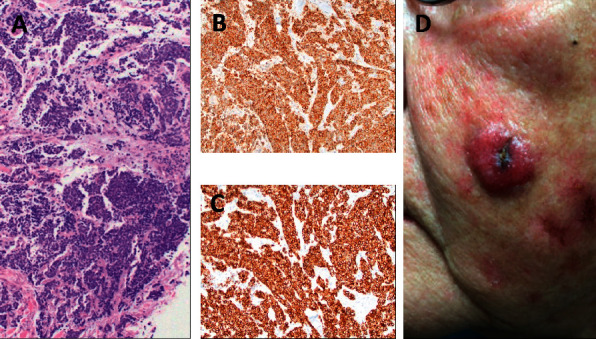
Shave biopsy pathology from the left cheek primary lesion. (a) Small purple cells consistent with neuroendocrine carcinoma. (b) Positivity for synaptophysin. (c) Perinuclear dot-like positivity for cytokeratin 20. (d) Primary lesion two weeks after initial confirmatory biopsy.

A 77-year-old Caucasian female presented for multidisciplinary consult at our tertiary care center with a left cheek nodule growing over 4-5 months with more recent rapid growth. Shave biopsy of a 14 mm firm nodule 2 months prior to presentation to our clinic had shown MCC extending to peripheral and deep tissue edges with perinuclear dot-like positivity for CK20, positivity for synaptophysin, weak focal reactivity for AE1/AE2, and negative staining for TTF-1 (Figures 1 and 2).

**Figure 3:**
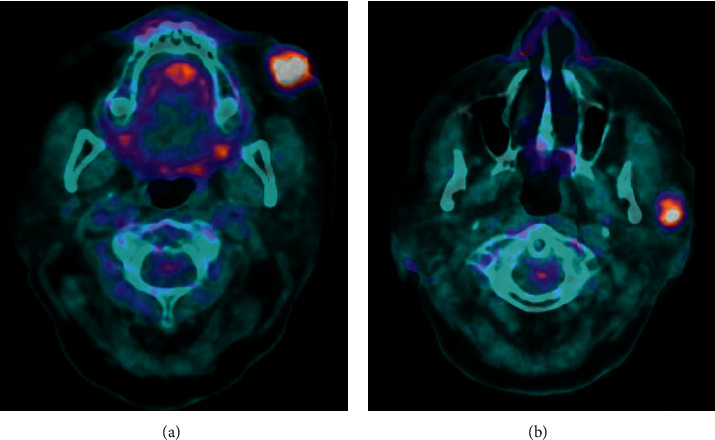
Staging PET/CT scan. (a) Primary site of disease to the left cheek. (b) Regional metastasis to the left parotid.

Outside hospital (OSH) staging PET/CT scan revealed focal intense FDG avidity in the primary left cheek mass, as well as focal activity in multiple intraparotid lymph nodes, with no evidence of distant disease (Figure 3). Past medical history was notable for hypertension, hyperlipidemia, depression, and degenerative joint disease; there was no prior history of skin cancer or immunosuppression. Family history was negative for melanoma or nonmelanoma skin cancers.

**Figure 4:**
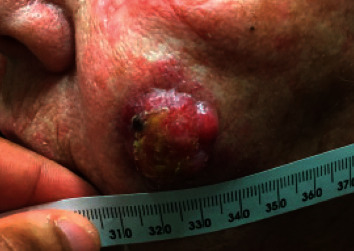
Primary site of disease to the left cheek (27 × 22 mm) at the time of presentation to our multidisciplinary clinic (7.5 weeks after confirmatory biopsy).

Our examination revealed an exophytic, friable tumor measuring 27 mm × 22 mm on the left cheek and palpable mass in the left parotid and submandibular areas (Figure 4). Vitals were notable for a blood pressure of 168/78. ECOG performance status was 1. Initial labs were unremarkable. Her anti-Merkel cell polyomavirus oncoprotein antibody titer (AMERK test, University of Washington) was negative. She underwent core biopsy of the left submandibular mass which confirmed MCC infiltrating the connective tissue, with overt lymphatic invasion. She was staged as pathological stage IIIB disease (T2pN1bM0). Targeted next-generation sequencing (NGS) of her tumor revealed variants in TP53, RB1, FLT3, and PDGFRA. Due to the multifocality of her disease on presentation, coupled with the poor prognosis of patients with stage IIIB disease, the decision was made to proceed with bimodality therapy. She was initiated on pembrolizumab (200 mg, IV, q3 weeks) and radiation.

One week after her first dose of pembrolizumab and prior to initiation of radiotherapy, she presented to OSH emergency department with sudden-onset abdominal pain and nausea. Complete metabolic panel, complete blood count, troponin, and lipase were all within normal limits. Inpatient gastroenterology consultation concluded that the abdominal symptoms were likely pembrolizumab-related. Her symptoms resolved, and she was released after 1 day of inpatient supportive care with IV fluids, ondansetron, and prochlorperazine. She went on to complete a radiotherapy course of 60 gray to the left cheek and 54 gray to the left neck.

After approximately 2 months, and 3 doses, of immunotherapy and 1.5 months after commencing radiotherapy, the patient complained of oropharyngeal discomfort and was prescribed fluconazole and clotrimazole troches at OSH radiation oncology clinic for possible thrush.

A few days later, she presented to an OSH emergency room and was ultimately admitted with nausea, vomiting, fatigue, and more acute symptoms of somnolence, slurred speech, and shortness of breath. At presentation, her labs were remarkable for transaminitis (ALT of 213 U/L, ref: 0–40 U/L; AST of 252 U/L, ref: 0–37 U/L), conjugated hyperbilirubinemia (total bilirubin of 4.5 mg/dL, ref: 0.0–1.2 mg/dL; direct bilirubin of 4.0 mg/dL, ref: 0.0–0.3 mg/dL; indirect bilirubin of 0.5 mg/dL; ref: 0.0–1.5 mg/dL), alkaline phosphate (ALP) elevation (535 U/L, ref: 39–117 U/L), and PT-INR elevation to 1.3 (ref: 0.9–1.1). Labs were additionally notable for high-sensitivity troponin T elevation (67 ng/L, ref: 0–9 ng/L), creatinine elevation (2.70 mg/dL, ref: 0.5–1.5 mg/dL), leukocytosis, and lactatemia. She was managed with empiric broad-spectrum antibiotics and intravenous fluids with concern for sepsis. Multiorgan toxicity from pembrolizumab was also high on the differential. Her electrocardiogram showed normal sinus rhythm and anterior Q waves; transthoracic echocardiogram showed a left ventricular ejection fraction of 61% with no evidence of wall motion abnormality or pericardial effusion. A right upper quadrant ultrasound showed hepatomegaly with heterogeneous increased echotexture suggestive of fatty infiltration and cholelithiasis but no focal hepatic lesions or evidence of biliary obstruction.

**Figure 5:**
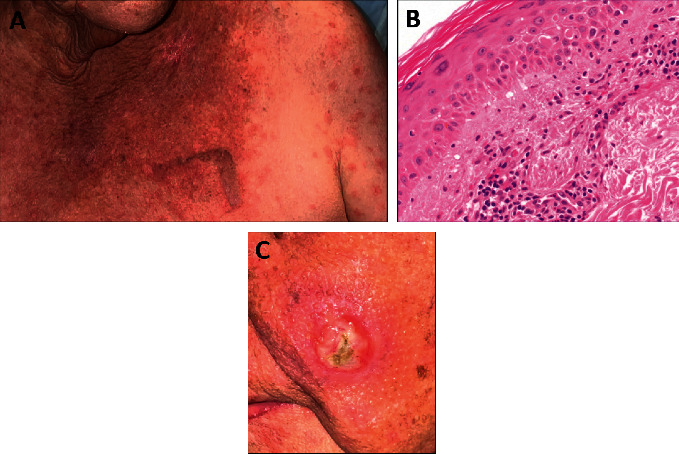
(a) Truncal eruption of pink macules and thin papules with minimal overlying scale coalescing centrally at the upper back and chest, with extension to the rest of torso and upper extremities, hospital day 4. (b) Biopsy revealed an interface dermatitis with dyskeratosis and superficial perivascular lymphocytic infiltrate. (c) Primary lesion to the left cheek showing significant recession on clinical examination after bimodality therapy, hospital day 4.

Renal function and lactate readily responded to intravenous fluids. However, her liver function at the OSH continued to worsen, and on hospital day 3, she was started on intravenous methylprednisolone 80 mg daily for presumed autoimmune hepatitis and possible autoimmune myocarditis. She was then transferred to our hospital. Viral and autoimmune hepatitis panels were unremarkable. On hospital day 4, she was noted to have developed a nonpruritic eruption concentrated on the upper chest and back and upper extremities. A punch biopsy revealed an interface dermatitis with dyskeratosis and superficial perivascular lymphocytic infiltrate (Figure 5).

**Figure 6:**
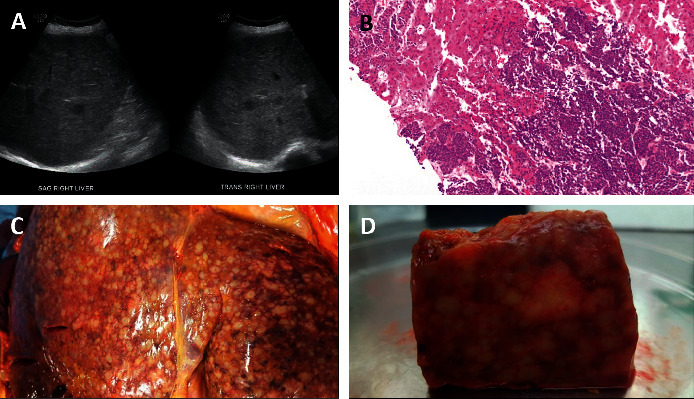
(a) Right upper quadrant ultrasound, hospital day 4, was notable for diffusely heterogeneous hepatic parenchyma with scattered hypoechoic lesions, suspicious for malignancy. No intrahepatic biliary ductal dilatation was observed. (b) Core liver biopsy revealed metastatic disease occupying roughly 60% of the total parenchyma. Background hepatic parenchyma was without signs of marked inflammation. (c) Gross inspection of the liver revealed replacement of the parenchyma by firm tan/yellow confluent nodules and punctate hemorrhagic areas. (d) Cross section of hepatic tissue recapitulated overwhelming tumor burden.

By hospital day 5, methylprednisolone was increased to pulse dose (1 g/day) in the setting of worsening bilirubinemia. High-sensitivity troponin T peaked at 143 ng/L before declining to 131 ng/L (ref: 0–9 ng/L), coinciding with escalation of pulse-dose steroids. Renal function precluded cardiac MRI, and coagulopathy was a relative contraindication for a myocardial biopsy. At this time, a repeat right upper quadrant ultrasound was obtained which was notable again for diffusely heterogeneous hepatic parenchyma, now interpreted as being suspicious for metastatic disease. An ultrasound-guided nonfocal liver biopsy was obtained, which confirmed extensive metastatic disease (Figures 6(a) and 6(b)). In the setting of progressive delirium and clinical decline, the patient was transitioned to symptom-directed therapy. She expired on hospital day 8.

At autopsy, 80% of the hepatic parenchyma was found to be replaced by tumor, with the remaining background tissue revealing steatosis, cholestasis, and necrosis with minimal inflammation (Figures 6(c) and 6(d)). Microscopic metastases were found in the left ventricular myocardium and the septum, the largest measuring 0.2 cm. Tumor cells were also found to be infiltrating representative sections of the bone marrow and psoas muscle. There was no evidence of autoimmune inflammation in any of the examined organs.

## 3. Discussion

We present here the case of a patient with regionally metastatic MCC treated with pembrolizumab and radiation, presenting acutely with symptoms and persistent lab abnormalities suggestive of checkpoint inhibitor therapy- (CIT-) related dermatitis, hepatitis, and myocarditis. This was managed in accordance with ASCO guidelines with high-dose systemic steroids [6]. Although a contemporaneous skin biopsy was consistent with a CIT-induced lichenoid eruption, liver biopsy, shortly prior to death, and autopsy were remarkable for diffuse replacement of liver parenchyma by disease, as well as metastatic deposits in the heart, skeletal muscle, and bone. Minimal signs of tissue inflammation were observed in those tissues, highlighting a novel circumstance in which metastatic MCC masqueraded as multiple distinct end-organ irAEs and presented concomitantly with confirmed irAE of the skin.

Though nonspecific on exam and histology, the patient's rash to the trunk and extremities was compatible with a pembrolizumab-mediated hypersensitivity irAE. 30–40% of patients treated with anti-PD-1 immunotherapy experience all-grade dermatologic irAEs, while the incidence of all-grade rash is 14–17% [7, 8]. The usual onset of rash is after the second cycle of therapy [7]. Anti-PD-1-mediated rash typically presents to the trunk or extremities as a maculopapular, often pruritic, eruption. While there is a wide range of possible histological presentations, the most commonly observed is an interface dermatitis, as was noted in this patient's case [7, 9, 10].

A retrospective study of 491 patients treated with pembrolizumab for solid organ tumors showed that 14.3% developed liver injury, as defined by lab abnormalities, at a median of 62 days from therapy initiation. Within this subset, only 29% of cases, 4.1% of the 491 cases overall, were adjudicated as likely to be drug-related on expert review (i.e., given Drug-Induced Liver Injury Network (DILIN) expert opinion scale scores of 1, definite, 2, highly likely, or 3, probable); most of the remainder were attributed to hepatic involvement by malignancy. The authors suggested an increased emphasis on contrast-enhanced cross-sectional scans and early biopsy in the setting of liver enzyme abnormalities as possible strategies to help reduce diagnostic uncertainty and avoid unnecessary empiric immunosuppression [11]. Proceeding with such a “prospectively obtained” liver biopsy in our case, where the patient could not undergo contrast-enhanced study due to acute kidney injury, might have expedited diagnosis of diffuse progression, prompting earlier goals of care discussion with the patient and family. In the setting of multiorgan insult, suspicion for hepatic metastasis in this case only developed when abdominal ultrasound was repeated at our institution with findings suspicious for malignancy. While neither conventional ultrasound nor noncontrast CT have been shown to be sensitive modalities for detection of hepatic metastases, interpretation at the outside hospital was likely complicated by the diffuse involvement of liver parenchyma, with absence of distinct focal lesions on ultrasound, as was noted in a case of MCC of the right lower eyelid diffusely metastatic to the liver reported in 2019 [12–15]. By the DILIN severity index definition, our patient's initial presentation would have been classified as moderate-severe (score 3; elevated ALT, ALP, bilirubin, and/or INR and hospitalization or prolongation of existing hospitalization). Labs were consistent with a cholestatic pattern of injury (*R* = 1.2). Although checkpoint inhibitors are more associated with the hepatocellular pattern of injury, there are multiple case reports of pembrolizumab inducing cholestatic injury, even after a single dose [11, 16, 17].

Multiple case reports and series have explored the histologic features associated with immunotherapy-related liver injury. Pathology for all of a series of seven patients treated with nivolumab or ipilimumab whose liver enzyme abnormalities responded to cessation of checkpoint inhibition and immunosuppression showed lobular hepatitis with milder portal inflammation [18]. Similarly, in another series of 8 patients treated with either pembrolizumab or nivolumab between 2016 and 2018 presenting with liver function abnormalities, again with clinical improvement on treatment cessation and immunosuppressive therapies, six out of eight cases were notable for histologic signs of acute lobular hepatitis, while 2 cases were characterized by varying degrees of steatotic and cholestatic changes [19]. Across both groups of patients, lobular scattered or spotty pattern necrosis was most frequently noted, although a few cases had more centrilobular and/or confluent patterns of necrosis. In contrast, liver histology for this case was remarkable for 80% replacement of hepatic parenchyma by tumor cells. The remaining parenchyma revealed nonspecific findings of patchy steatosis, cholestasis, and centrilobular necrosis with minimal inflammation. Significantly, the patient had already been initiated on steroids before tissue biopsy was performed. While the time course of histologic response of immune-related hepatitis to immunosuppression is not well characterized, a case report of pembrolizumab-induced cholestatic liver injury showed biopsy evidence of continued inflammation of the portal tract despite prior initiation of steroids [16].

Myocarditis relating to CIT has an all-grade reported incidence ranging from 0.04% to 1.14%, with a mortality rate of up to 50% [20–22]. Cases of CIT-related myocarditis typically occur within 1-2 months of therapy initiation and may develop even after a single dose [22, 23]. This patient presented with presumed pembrolizumab-related myocarditis, ASCO grade 1-2 (abnormal cardiac biomarkers and electrocardiogram, possibly with mild symptoms) [6, 24]. EKG findings relating to myocarditis are diverse and nonspecific. While T-wave changes are the most common finding, Q-waves, as in this case, have also been documented [23, 25–27]. Echocardiogram for this patient was unremarkable; further cardiac workup was limited by renal and liver dysfunction. Ultimately, diagnosis of myocardial metastases was made on autopsy with no signs of inflammation in examined cell blocks, again with caveat of exposure to high-dose steroids during admission.

While infrequent overall, since 2001, many cases of cardiac metastasis of MCC have been reported in the literature, with clinical consequences including symptoms of chest pain and shortness of breath, various degrees of heart block, and malignant pericardial effusion with resultant tamponade [28–33]. With an additional complication of acute kidney injury, the scattered cardiac metastases observed on autopsy probably drove the acute hypertroponinemia observed in this patient's case, as has been observed in other head and neck cancers metastatic to the heart. A case of a 25-year-old woman with lingual squamous cell carcinoma with cardiac metastasis was notable for elevation in troponin-I and ST-elevation in leads V3–V6 attributed to metastatic infiltration of the myocardium rather than ischemic disease, although the case featured a much larger deposit of disease extending from the right ventricular free wall [34]. On-target toxicity from CIT may also have contributed. A 65-year-old woman treated with a single dose of nivolumab and experimental drug for stage IV squamous cell carcinoma of the lung presented with signs of myocarditis, including troponin-I elevation (0.12 ng/mL), LVEF of 25–30%, and enhancement on cardiac MRI, and was eventually discharged after improvement with immunosuppression. The patient later developed cardiogenic shock and was transitioned to comfort care in the setting of newly discovered hemorrhagic stroke. Notably, autopsy revealed widely metastatic squamous cell carcinoma, including metastatic deposits in both ventricles, the septum, and the right atrium [35].

## 4. Conclusions

Merkel cell carcinoma is an aggressive neuroendocrine cancer of the skin, with great propensity for regional and distant metastasis. Multisite progression of disease may mimic simultaneous presentation of multiple immune-related adverse events, especially if options for further workup are limited by clinical contraindications. Early diagnosis of progression may help avoid unnecessary immunosuppression and expedite curative or palliative therapy.

## Abbreviations

ALP: Alkaline phosphatase
ALT: Alanine transaminase
AMERK: Merkel polyomavirus serology test
ASCO: American Society of Clinical Oncology
AST: Aspartate transaminase
CK: Cytokeratin
CT: Computed tomography
DILIN: Drug-Induced Livery Injury Network
DNR/DNI: Do not resuscitate/do not intubate
ECOG: Eastern Cooperative Oncology Group
EKG: Electrocardiogram
FDA: The United States Food and Drug Administration
FDG: Fluorodeoxyglucose
INR: International normalized ratio
IrAE: Immune-related adverse event
LFTs: Liver function tests
LVEF: Left ventricular ejection fraction
MCC: Merkel cell carcinoma
MRI: Magnetic resonance imaging
NCDB: National Cancer Database
NGS: Next-generation sequencing
OSH: Outside hospital
PET: Positron emission tomography
PT: Prothrombin time
US: Ultrasound
CIT: Checkpoint inhibitor therapy.

## References

[1] Lemos B. D., Storer B. E., Iyer J. G. (2010). Pathologic nodal evaluation improves prognostic accuracy in Merkel cell carcinoma: analysis of 5823 cases as the basis of the first consensus staging system. *Journal of the American Academy of Dermatology*.

[2] Harms K. L., Healy M. A., Nghiem P. (2016). Analysis of prognostic factors from 9387 Merkel cell carcinoma cases forms the basis for the new 8th edition AJCC staging system. *Annals of Surgical Oncology*.

[3] Tello T. L., Coggshall K., Yom S. S., Yu S. S. (2018). Merkel cell carcinoma: an update and review. *Journal of the American Academy of Dermatology*.

[4] Kouzmina M., Koljonen V., Leikola J., Böhling T., Lantto E. (2017). Frequency and locations of systemic metastases in Merkel cell carcinoma by imaging. *Acta Radiologica Open*.

[5] Health N. I. O. (2017). Livertox: clinical and research information on drug-induced liver injury.

[6] Brahmer J. R., Lacchetti C., Schneider B. J. (2018). Management of immune-related adverse events in patients treated with immune checkpoint inhibitor therapy: American society of clinical oncology clinical practice guideline. *Journal of Clinical Oncology*.

[7] Naidoo J., Page D. B., Li B. T. (2015). Toxicities of the anti-PD-1 and anti-PD-L1 immune checkpoint antibodies. *Annals of Oncology*.

[8] Lewis C. W., Qazi J., Hippe D. S. (2020). Patterns of distant metastases in 215 Merkel cell carcinoma patients: implications for prognosis and surveillance. *Cancer Medicine*.

[9] Belum V. R., Benhuri B., Postow M. A. (2016). Characterisation and management of dermatologic adverse events to agents targeting the PD-1 receptor. *European Journal of Cancer*.

[10] Wang D. Y., Johnson D. B., Davis E. J. (2018). Toxicities associated with PD-1/PD-L1 blockade. *The Cancer Journal*.

[11] Tsung I., Dolan R., Lao C. D. (2019). Liver injury is most commonly due to hepatic metastases rather than drug hepatotoxicity during pembrolizumab immunotherapy. *Alimentary Pharmacology & Therapeutics*.

[12] Kinkel K., Lu Y., Both M., Warren R. S., Thoeni R. F. (2002). Detection of hepatic metastases from cancers of the gastrointestinal tract by using noninvasive imaging methods (US, CT, MR imaging, PET): a meta-analysis. *Radiology*.

[13] Jee H. B. (2015). Is non-contrast CT adequate for the evaluation of hepatic metastasis in patients who cannot receive iodinated contrast media?. *PLoS One*.

[14] Zwiebel W. J. (1995). Sonographic diagnosis of diffuse liver disease. *Seminars in Ultrasound, CT and MRI*.

[15] Ana L. S. (2020). A rare cause of acute liver failure: diffuse liver metastization of Merkel cell carcinoma. *GE-Portuguese Journal of Gastroenterology*.

[16] Kurokawa K., Hara M., Iwakami S.-I. (2019). Cholestatic liver injury induced by pembrolizumab in a patient with lung adenocarcinoma. *Internal Medicine*.

[17] Penn J. R., Rustgi V. (2017). An unusual case of cholestatic drug-induced liver injury following a single dose of pembrolizumab. *American Journal of Gastroenterology*.

[18] Zen Y., Yeh M. M. (2018). Hepatotoxicity of immune checkpoint inhibitors: a histology study of seven cases in comparison with autoimmune hepatitis and idiosyncratic drug-induced liver injury. *Modern Pathology*.

[19] Zhang D. (2019). Histologic patterns of liver injury induced by anti-PD-1 therapy. *Gastroenterology Report*.

[20] Mahmood S. S., Fradley M. G., Cohen J. V. (2018). Myocarditis in patients treated with immune checkpoint inhibitors. *Journal of the American College of Cardiology*.

[21] Al-Kindi S. G., Oliveira G. H. (2018). Reporting of immune checkpoint inhibitor-associated myocarditis. *The Lancet*.

[22] Moslehi J. J., Salem J.-E., Sosman J. A., Lebrun-Vignes B., Johnson D. B. (2018). Increased reporting of fatal immune checkpoint inhibitor-associated myocarditis. *The Lancet*.

[23] Zhang L., Awadalla M, Mahmood S. S (2020). Cardiovascular magnetic resonance in immune checkpoint inhibitor-associated myocarditis. *European Heart Journal*.

[24] Palaskas N. (2020). Immune checkpoint inhibitor myocarditis: pathophysiological characteristics, diagnosis, and treatment. *Journal of the American Heart Association*.

[25] Upadhrasta S., Elias H., Patel K., Zheng L. (2019). Managing cardiotoxicity associated with immune checkpoint inhibitors. *Chronic Diseases and Translational Medicine*.

[26] Nakashima H., Honda Y., Katayama T. (1994). Serial electrocardiographic findings in acute myocarditis. *Internal Medicine*.

[27] Deluigi C. C., Ong P., Hill S. (2013). ECG findings in comparison to cardiovascular MR imaging in viral myocarditis. *International Journal of Cardiology*.

[28] Conley M., Hawkins K., Ririe D. (2006). Complete heart block and cardiac tamponade secondary to Merkel cell carcinoma cardiac metastases. *Southern Medical Journal*.

[29] Kazemi N. Y., Jain C., Bois M. C., Behfar A., Olivier K., Markovic S. N. (2019). Heart block caused by cardiac metastasis from Merkel cell carcinoma: a case report. *Mayo Clinic Proceedings: Innovations, Quality & Outcomes*.

[30] Di Loreto M., Francis R. (2017). Merkel cell carcinoma cardiac metastasis causing cardiac tamponade. *BMJ Case Reports*.

[31] Yamana N., Sueyama H., Hamada M. (2004). Cardiac metastasis from Merkel cell skin carcinoma. *International Journal of Clinical Oncology*.

[32] Page E. (2001). Cardiac metastasis from a Merkel cell skin carcinoma. *A case report. Archives des maladies du coeur et des vaisseaux*.

[33] Suttie C. F., Hruby G., Horvath L., Thompson J. (2014). Cardiac metastasis in Merkel cell carcinoma. *Journal of Clinical Oncology*.

[34] Patel H., Francke M., Stahura H., El-Hajjar M., Schulman-Marcus J. (2018). Solitary cardiac metastasis from primary oral squamous cell carcinoma presenting as ST-elevation MI. *BMJ Case Reports*.

[35] Agrawal N., Khunger A., Vachhani P. (2019). Cardiac toxicity associated with immune checkpoint inhibitors: case series and review of the literature. *Case Reports in Oncology*.

